# Modified gegen qinlian decoction ameliorates *Veillonella parvula*-exacerbated ulcerative colitis via restoration of intestinal mucosal barrier function

**DOI:** 10.3389/fphar.2026.1803816

**Published:** 2026-04-21

**Authors:** Meiling She, Xinyi Dai, Xintong Wang, Zheng Wang, Jiaqi Zhang, Zhuo Shi, Ying Peng, Xiaobo Li, Ting Chen, Xudong Tang

**Affiliations:** 1 Institute of Digestive Diseases, Xiyuan Hospital of China Academy of Chinese Medical Sciences, Beijing, China; 2 China Academy of Chinese Medical Sciences, Beijing, China; 3 School of Pharmacy, Shanghai Jiao Tong University, Shanghai, China

**Keywords:** intestinal barriers, mechanical barrier, mucus barrier, ulcerative colitis, Veillonella parvula

## Abstract

**Introduction:**

Ulcerative colitis (UC) is being increasingly connected to the pathogen *Veillonella parvula* (V. parvula). Modified Gegen Qinlian Decoction (MGQD), a traditional Chinese medicine therapy, is used to treat UC. However, the underlying mechanism by which V. parvula exacerbates UC and whether MGQD can alleviate it is unknown.

**Methods:**

MGQD therapy was tested on pathogen-free and pseudo-germ-free mice infected with V. parvula. Colitis severity was determined by disease activity index, colon length, and histology. Transmission electron microscopy, Western blot, immunofluorescence, and immunohistochemistry were used to assess the integrity of the intestinal mucosal barrier. *In vitro*, mouse colonic organoids were utilized to examine the effects of baicalin and puerarin on inflammation-induced downregulation of Muc2 and ZO-1.

**Results:**

MGQD therapy dramatically reduced the severity of colitis in both mouse models. MGQD restored the integrity of the mucus and mechanical barriers. Furthermore, baicalin and puerarin reduced inflammation-induced downregulation of Muc2 and ZO-1 in mouse colonic organoids.

**Discussion:**

These findings demonstrate that MGQD alleviates intestinal barrier dysfunction caused by V. parvula, providing novel mechanistic insight for the management of chronic colitis.

## 1 Introduction

Ulcerative colitis (UC), a chronic relapsing inflammatory bowel disease characterized by persistent colonic and rectal inflammation, presents with hallmark symptoms including hematochezia, abdominal pain, and tenesmus, which significantly impair patient quality of life (Wangchuk et al., 2024). With rapidly rising incidence in middle- and low-income regions, the global burden of UC continues to escalate while clinical management becomes increasingly complex (Ng et al., 2017; Kontola et al., 2023).

UC pathogenesis involves genetic susceptibility, impaired intestinal barrier function, immune dysregulation, and environmental triggers (Xu et al., 2024). An early critical event is the structural weakening of the colonic mucus barrier, which compromises defense against pro-inflammatory stimuli from the gut microbiota (van der Post et al., 2019). Concurrent dysfunction of tight junctions, which regulated by proteins such as Occludin and ZO-1, increases epithelial permeability and disrupts mucosal homeostasis (Vivinus-Nébot et al., 2014). This collapse of barrier integrity promotes pathogenic microbial translocation, sustaining mucosal inflammation and tissue injury (Liang et al., 2024).

UC is also fundamentally associated with gut microbial dysbiosis, marked by reduced commensal diversity and expansion of pro-inflammatory taxa (Zuo and Ng, 2018). Notably, *Veillonella parvula* (*V. parvula*), often enriched in UC patients, exacerbates inflammation via TLR4-dependent macrophage activation, degradation of mucin layers, and suppression of anti-inflammatory butyrate production (Patel et al., 2022; Zhan et al., 2022). During active inflammation, nitrate respiration further enhances its colonization and ecological fitness (Rojas-Tapias et al., 2022; Le Berre et al., 2023). Thus, mucosal barrier breakdown and microbial dysbiosis form a vicious cycle that drives UC progression, making barrier restoration and microbiota modulation key therapeutic goals.

Current management of UC relies on four main drug classes: aminosalicylates, corticosteroids, immunomodulators, and biologics. Aminosalicylates and corticosteroids serve as first-line treatments for mild-to-moderate UC, whereas moderate-to-severe cases often require immunomodulators or biologics, frequently used in combination. However, these therapies are often limited by steroid dependency, dose-related toxicities, and inconsistent treatment responses.

Growing evidence indicates that Traditional Chinese Medicine (TCM) compounds can modulate intestinal immunity by reshaping the gut microbiota, exerting multi-target synergistic effects in UC treatment (Liu et al., 2022). Among them, Modified Gegen Qinlian Decoction (MGQD), which is an optimized formulation based on the classic prescription, clinical experience, and dampness-heat syndrome theory, has shown enhanced efficacy. Our preliminary studies demonstrated that MGQD alleviates colitis symptoms and reduces colonic damage in murine UC models (Huang et al., 2024; Wang et al., 2023). Nevertheless, the precise mechanisms underlying its therapeutic effect remain unclear.

Therefore, this study aims to elucidate the therapeutic mechanisms of MGQD in UC by investigating its precision-targeted modulation of *V. parvula* and subsequent restoration of dual-barrier integrity and specifically, the intestinal physical barrier and mucus layer.

## 2 Materials and methods

### 2.1 Chemicals and reagents

Dextran sulfate (DSS) was obtained from Meilunbio (Dalian, China, Batch number:MB5535). Hematoxylin and eosin (H&E) and Alcian Blue/Periodic Acid-Schiff (AB/PAS) staining kits were provided by Servicebio (Wuhan, China, Batch number:G1049). Antibodies against ZO-1 was provided by Invitrogen (CA, United States, Batch number:617033). Antibodies against E-cadherin (Batch number: ab76319) and δ-catenin (Batch number: ab184917) were purchased from Abcam (Waltham, MA, United States). Antibodies against Occludin (Batch number:GB111401), Muc2 (Batch number:GB11344) and GAPDH (Batch number:GB15002) were provided by Proteintech (Wuhan, China). The enzyme-linked immunosorbent assay (ELISA) detection kits for the cytokines IL-1β,IL-6 and TNF-α were purchased from the Mlbio (Shanghai, China). *Veillonella parvula* (ATCC 10790) was provided by Testobio (Ningbo, China). The enhanced BCA protein assay kit (Batch number:P0010) was get from Beyotime (Shanghai, China). The total protein extraction maxi kit (Batch number:BC3710) was purchased Solarbio (Beijing, China). Puerarin (3681-99-0) and baicalin (21967-41-9) were obtained from Shanghai Yanye Biological Technology Co., Ltd. (Shanghai, China). TNF-α were obtained from Thermo Fisher (315-01A).

### 2.2 Preparation of MGQD

MGQD is composed of *Pueraria lobata (Willd.) Ohwi*, *Scutellaria baicalensis Georgi*, *Coptis chinensis Franch.*, *Euphorbia humifusa* Willd. ex Schltdl., Zingiber offcinale Rose., and *Glycyrrhiza uralensis Fisch.*. Raw medicinal plants were supplied by Xiyuan Hospital, Chinese Academy of Chinese Medical Sciences (Beijing, China) and processed according to our previously described protocols (Wang et al., 2023; Wang et al., 2021). The production process of MGQD extract was as follows: (a) Talcum was cooked in water for 30 min, followed by the addition of the remaining herbs and boiling for 1.5 h before filtration; (b) the filtrate was then mixed with six times distilled water and boiled for 1 h before filtering again; (c) the resulting filtrate was combined to produce crude drug at a concentration of 1.87 g/mL.

### 2.3 Chemical components and quality control of MGQD

To determine the primary chemical components of MGQD, UPLC-QTOF-MS/MS was employed. An Agilent 1290 UPLC system (United States) with a ZORBAX Eclipse Plus C18 column (2.1 × 100 mm, 1.8 μm, Agilent Ltd., United States) was used for chromatographic analysis. An Agilent 6545 QTOF spectrometer with a Dual AJS ESI source (Agilent Ltd., United States) in both positive and negative ion modes was used to conduct mass spectrometry. The Supplementary file displays the mobile phase and specific detection parameters.

### 2.4 Animals and experimental protocol

Male mice were obtained from Charles River Laboratories (license number: SCXK (Jing) 2021-0006). The authors have adhered to the ARRIVE guidelines (https://arriveguidelines.org/). The mice were acclimatized and fed for 1 week at 25 °C ± 1 °C and relative humidity of 55% ± 5%. The status of the mice (body weight, mental and behavioral conditions, etc.) was observed daily, and timely interventions were taken according to the status of the mice to ensure animal welfare. Following acclimation, 36 male C57BL/6J mice (18–22 g) were randomly divided into six groups (n = 6): Control (CON), DSS model (DSS), DSV, low-dose MGQD (DSGL; 4.485 g/kg), medium-dose MGQD (DSGM; 8.97 g/kg), and high-dose MGQD (DSGH; 17.94 g/kg). Acute UC was induced in all non-CON groups using 3% (w/v) DSS in drinking water for 7 days. On days 2, 4, and 6, all groups except CON and DSS received oral gavage of Veillonella parvula (1 × 10^9^ CFU), while CON, DSS, and DSV groups received equivalent volumes of vehicle (distilled water). Daily monitoring included body weight, stool consistency, diarrhea, and hematochezia. All mice were euthanized on day 10; colons were immediately excised for length measurement, and tissues were snap-frozen in liquid nitrogen for storage at −80 °C pending analysis. All the experiments were approved by the animal ethics committee of Xiyuan Hospital, China Academy of Chinese Medical Sciences (Approval NO. 2025XLC010).

### 2.5 Disease activity index

Body weight, fecal consistency, and blood stools were measured daily in mice, and DAI scores were calculated according to previously reported methods (Huang et al., 2024; Kihara et al., 2003).

### 2.6 Histological assessment

To assess histological changes, distal colon segments (1.5 cm) were fixed in 4% paraformaldehyde for 48 h, paraffin-embedded, sectioned (4 μm), and stained with H&E or AB/PAS following established protocols. Histopathological scoring was performed according to published criteria (Huang et al., 2024; Wei et al., 2021).

### 2.7 Immunohistochemistry and immunofluorescence evaluation

For immunohistochemistry (IHC), fresh colon tissues were fixed in 4% paraformaldehyde, paraffin-embedded, and sectioned (4 μm). Sections were incubated overnight at 4 °C with primary antibodies against ZO-1 and Occludin followed by biotinylated secondary antibodies. Stained slides were imaged using light microscopy (She et al., 2025).

For immunofluorescence (IF), dewaxed sections were blocked with 3% BSA/PBS (30 min), incubated with anti-Muc2 antibody, and detected with appropriate secondary antibodies. Nuclei were counterstained with DAPI prior to mounting in antifade medium. Muc2 expression was visualized by fluorescence microscopy (She et al., 2025).

### 2.8 Intestinal barrier observation via transmission electron microscopy

Colon tissues were dissected into 1 mm^3^ fragments and fixed overnight in 2.5% glutaraldehyde at 4 °C. After PBS washes, samples were post-fixed in 1% osmium tetroxide (2 h), dehydrated through an ethanol series, and embedded in epoxy resin. Ultrathin sections (70 nm) were stained with 2% uranyl acetate (1 h) and imaged using a JEM1400 TEM (Jeol, Tokyo Japan) (Huang et al., 2024).

### 2.9 Cytokine detection

Serum levels of IL-1β, IL-6, and TNF-α were quantified using ELISA kits per manufacturer’s protocol. In brief, the pre-coated plate was blocked. Subsequently, samples, standards, and controls were added and incubated at 37 °C. After washing, biotinylated detection antibody was added, followed by incubation with enzyme conjugate. Tetramethylbenzidine substrate was used for color development, and the reaction was stopped with 2 M sulfuric acid. The optical density was measured at 450 nm using a microplate reader.

### 2.10 Western blotting assay

Total protein was extracted from colon tissues using RIPA buffer supplemented with protease inhibitors and quantified via BCA assay. Equal protein amounts were resolved on 10% SDS-PAGE gels and transferred to PVDF membrane. After blocking with 5% skim milk (1 h), membranes were incubated overnight at 4 °C with primary antibodies against ZO-1, Occludin, E-cadherin, δ-catenin, and GAPDH. Following 1 h incubation with HRP-conjugated secondary antibodies, protein bands were visualized by enhanced chemiluminescence and quantified using ImageJ software. All primary antibodies were diluted at a ratio of 1:1000 in a dedicated antibody diluent. Subsequently, the secondary antibodies were diluted 1:1000 in TBST.

### 2.11 Metagenome sequencing

Metagenomic libraries were prepared from 100 ng DNA (TruSeq Nano DNA Library Prep Kit, Illumina), size-selected (∼350 bp), and sequenced on Illumina MiSeq. Raw FASTQ files were quality-trimmed using Trimmomatic (v0.36). Valid reads were assembled with MEGAHIT (v1.1.2), and scaffolds were fragmented at gaps into Scaftigs (≥500 bp retained). Open reading frames (ORFs) were predicted with Prodigal (v2.6.3) and translated. Non-redundant gene catalogs were generated using CD-HIT (v4.6.7; 95% identity, 90% coverage), with the longest sequence representing each cluster. Sample reads were mapped to this catalog via Bowtie2 (v2.2.9; 95% identity) for gene abundance quantification. Representative sequences were functionally annotated against NR, KEGG, COG, Swiss-Prot, and GO databases (e-value ≤1e-5). Taxonomic profiling was derived from NR taxonomy, with abundances aggregated across all standard ranks (domain to species).

### 2.12 Establishment and treatment of mouse colon organoids

From the cecum to the anus, the whole colon was removed. Feces and mucus were carefully scraped off using a microscope slide after the colon was cut longitudinally. After the colon was sliced into 2 mm pieces, impurities were thoroughly cleaned with PBS. Colon pieces were incubated for 30 min at 4 °C in PBS with 10 mM EDTA (Solarbio, E1170) and antibiotics to release the crypts. After that, the crypts were filtered through a 100 μm sieve to get rid of tissue pieces and villus debris. After counting the isolated crypts, 50 μL of Matrigel (Corning, 356234) were added to each 300–500 crypts and plated in 24-well plates. Mouse Colon Organoid Culture Medium (Shanghai JFKR Organoid Biotechnology Co. Ltd., JFKR-MNC-100) was used to cultivate crypts at 37 °C in a humidified 5% CO2 environment. Culture medium was replaced every 2–3 days.

### 2.13 Statistical analysis

Statistical analyses were performed using GraphPad Prism 88. Inter-group comparisons utilized two-tailed Student's t-tests (unequal variances). One-way ANOVA with Tukey’s *post hoc* test compared multiple groups under single variables. Non-normally distributed data were analyzed by Kruskal–Wallis test followed by Dunn’s *post hoc* comparison. Significance levels: *P < 0.05, **P < 0.01, ***P < 0.001. Experimental statistical details appear in figure legends. Investigators were unblinded during group allocation and experimentation.

## 3 Results

### 3.1 MGQD alleviates the enrichment of *Veillonella parvula* in mice with a full microbiome under inflammatory conditions

**FIGURE 1:**
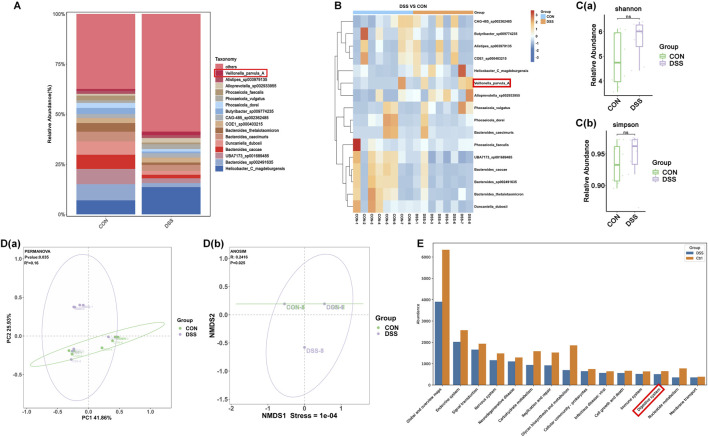
*Veillonella parvula* (*V. parvula*) enrichment in DSS-Induced colitis mouse model. **(A)** Column plot of species abundance of each sample at Species level. **(B)** Heatmap of species abundance of each sample at Species level. **(C)** The shannon index and simpson index. **(D)** The PCoA analysis and NMDS analysis. **(E)** The KEGG analysis of divergent microbiota. Group definitions: CON: Normal control mice. DSS: DSS-induced colitis mice. Data are expressed as mean ± SEM (n = 8).

Consistent with prior evidence linking gut inflammation to *Veillonella* enrichment, *V. parvula* are implicated in IBD progression (Zhan et al., 2022). To investigate *V. parvula* abundance dynamics in UC, we employed a DSS-induced murine model. *V. parvula* levels were significantly elevated in colonic luminal contents of DSS-treated mice versus controls (Figures 1A,B). DSS administration reduced gut microbiota β-diversity (Figure 1D) without altering α-diversity (Figure 1C). Functional analysis of differentially abundant bacteria revealed strong enrichment in pathways associated with digestive system (Figure 1E).

**FIGURE 2:**
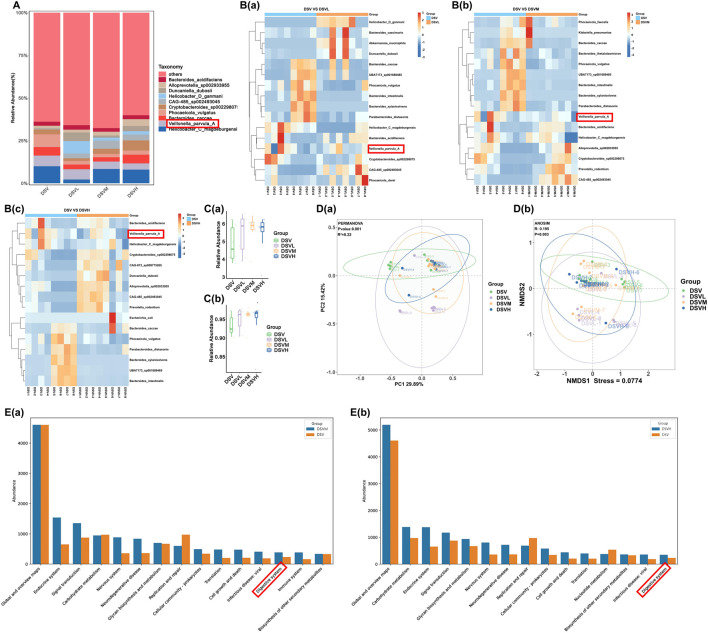
MGQD suppresses *V. parvula* enrichment in DSS-Induced colitis mice. **(A)** Column plot of species abundance of each sample at Species level. **(B)** Heatmap of species abundance of each sample at Species level. **(C)** The shannon index and simpson index. **(D)** The PCoA analysis and NMDS analysis. **(E)**The KEGG analysis of divergent microbiota. Data are expressed as mean ± SEM (n = 8). DSV: DSS + V. parvula intervention. DSVM: DSS + V. parvula + medium-dose MGQD. DSVH: DSS + V. parvula + high-dose MGQD. Data are expressed as mean ± SEM (n = 8).

To determine MGQD’s effect on *V. parvula* in DSS-Induced colitis mice, we quantified *V. parvula* abundance following graded MGQD administration. MGQD significantly reduced *V. parvula* colonization in DSS-induced colitis mice in a dose-dependent manner (Figures 2A,B). While α-diversity remained unaffected (Figure 2C), β-diversity increased significantly (Figure 2D). Functional analysis revealed differential bacterial enrichment pathways: low-dose MGQD showed no impact on gastrointestinal pathologies (Supplementary Figure S1D), whereas medium and high doses substantially altered digestive system-related functions (Figure 2E).

### 3.2 MGQD mitigates *Veillonella parvula*-Exacerbated colitis symptoms in mice with a full microbiome

**FIGURE 3:**
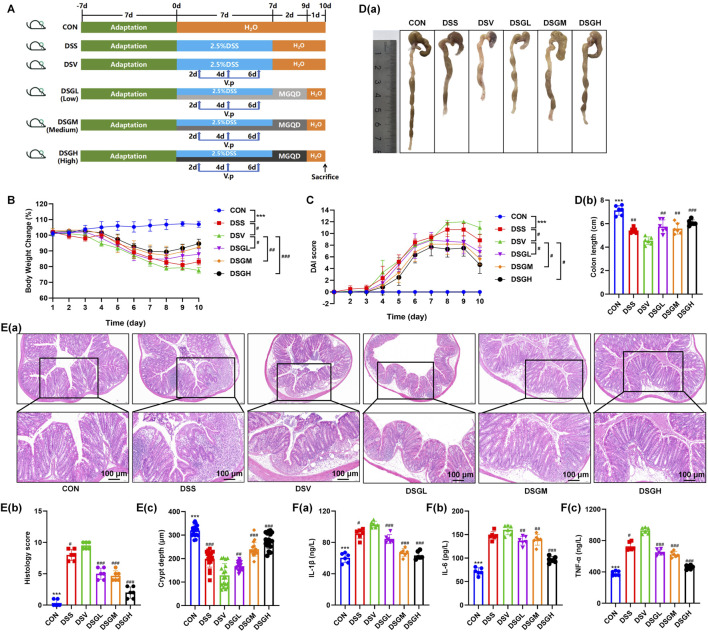
*V. parvula* aggravates DSS-induced colitis in mice, while the Modified Gegen Qinlian Decoction (MGQD) mitigates this exacerbation. **(A)** Experimental timeline. **(B)** Body weight changes in mice. **(C)** Disease Activity Index (DAI) scores. **(D)** Representative colon tissue images and corresponding quantitative analysis. **(E)** Hematoxylin and eosin (H&E) staining of colon tissues and histopathological scoring. **(F)** Levels of inflammatory cytokines in mouse serum. Data are presented as mean ± SEM. Group definitions: DSV: DSS + *V. parvula* intervention. DSGL: DSS + *V. parvula* + low-dose MGQD. DSGM: DSS + *V. parvula* + medium-dose MGQD. DSGH: DSS + *V. parvula* + high-dose MGQD. The data are expressed as the means ± standard deviation (S.D.)(n = 6). #P < 0.05, ##P < 0.01, ###P < 0.001 vs. the DSV group. *P < 0.05, **P < 0.01, ***P < 0.001 vs. the DSS group.

This study demonstrates that *V. parvula* significantly exacerbates disease progression in DSS-induced UC under inflammatory conditions (Figure 3A). Compared to the DSS-only control group, mice supplemented with *V. parvula* (DSV group) exhibited markedly reduced body weight (Figure 3B), elevated disease activity index scores (Figure 3C), significant colon shortening (Figure 3D), aggravated inflammation with crypt structural damage in histopathological analysis (Figure 3E), and increased serum levels of IL-1β, IL-6, and TNF-α, confirming enhanced systemic inflammation (Figure 3F). Conversely, MGQD intervention effectively reversed *V. parvula*-mediated disease exacerbation. In mice colonized with the bacteria, MGQD significantly increased body weight (Figure 3B), reduced disease activity index scores (Figure 3C), alleviated colon shortening (Figure 3D), mitigated mucosal damage by restoring crypt structure and increasing crypt depth (Figure 3E), and lowered serum levels of inflammatory cytokines (Figure 3F). Together, these findings confirm the therapeutic efficacy of MGQD against *V. parvula*-potentiated colitis.

### 3.3 MGQD ameliorates *Veillonella parvula*-Exacerbated mucus barrier damage in mice with a full microbiome

**FIGURE 4:**
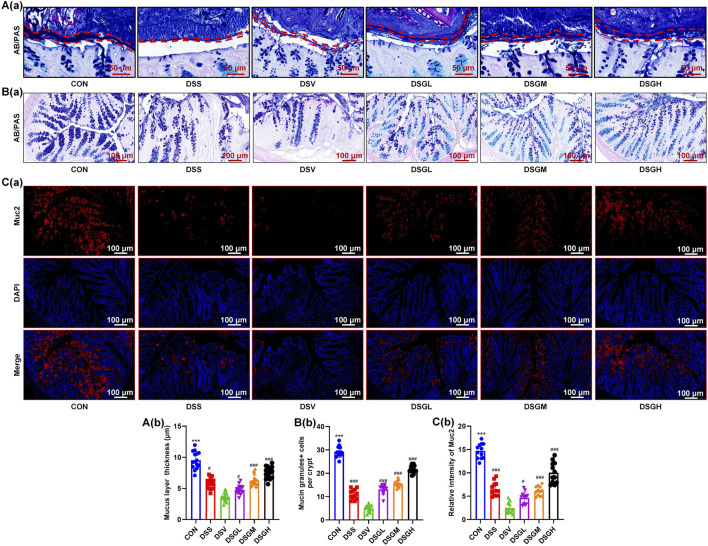
*V. parvula* exacerbates colonic mucus barrier damage in DSS-induced colitis mice, while the MGQD ameliorates this *V. parvula*-exacerbated damage. **(A,B)** AB-PAS staining in mouse colon. **(C)** The IF staining of mouse colon for Muc2 (red) and DAPI (blue). Data are expressed as mean ± S.D. (n = 6). #P < 0.05, ##P < 0.01, ###P < 0.001 vs. the DSV group. *P < 0.05, **P < 0.01, ***P < 0.001 vs. the DSS group.

To investigate the mechanistic role of *V. parvula* in promoting UC progression under inflammatory conditions, we examined its impact on the colonic mucus barrier in DSS-treated mice and found that it significantly exacerbates mucus barrier damage during inflammation, manifested by reduced mucus layer thickness (Figure 4A) and decreased goblet cell density (Figure 4B). Immunofluorescence analysis further confirmed that *V. parvula* mediates downregulation of mucin Muc2 expression (Figure 4C), indicating impaired mucus production as a key pathological mechanism. Based on these findings, we further investigated whether MGQD could alleviate *V. parvula*-induced mucus barrier disruption. The results showed that MGQD treatment significantly restored mucus layer thickness (Figure 4A) and increased goblet cell density in a dose-dependent manner (Figure 4B). Immunofluorescence analysis confirmed that MGQD prevented *V. parvula*-induced downregulation of Muc2 (Figure 4C), demonstrating its ability to maintain mucin production during inflammation.

### 3.4 MGQD restores *Veillonella parvula*-Exacerbated colonic mechanical barrier integrity in mice with a full microbiome

**FIGURE 5:**
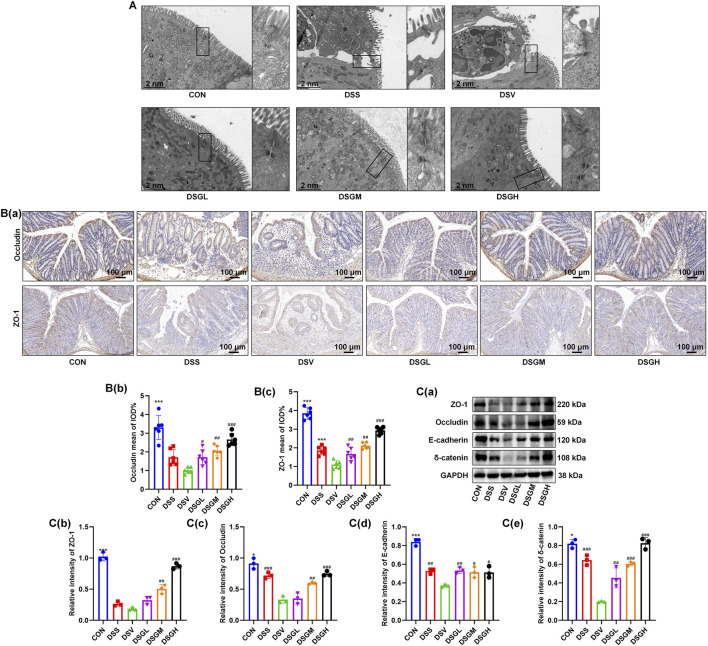
*V. parvula e*xacerbates colonic mechanical barrier damage in DSS-Induced colitis mice, while the MGQD ameliorates this *V. parvula*-exacerbated damage. **(A)** Transmission electron microscopy of mouse colon tissue. **(B)** The IHC staining in mouse colon. **(C)** The Western blot of mouse colon. Data are expressed as mean ± SEM (n = 6). The data are expressed as the means ± S.D. (n = 6). #P < 0.05, ##P < 0.01, ###P < 0.001 vs. the DSV group. *P < 0.05, **P < 0.01, ***P < 0.001 vs. the DSS group.

To further investigate the impact of *V. parvula* on the colonic mechanical barrier under inflammatory conditions, we examined its effects in DSS-treated mice. Our findings revealed that *V. parvula* significantly exacerbates mechanical barrier damage during inflammation, characterized by ultrastructural disruption of tight junctions and disorganized cellular adhesion architecture (Figure 5A), as well as a marked reduction in the expression of mechanical barrier biomarkers E-cadherin and δ-catenin (Figure 5B). Transmission electron microscopy further demonstrated that *V. parvula* notably aggravated DSS-induced tight junction disruption (Figure 5A). Immunohistochemical analysis showed that the expression of barrier markers ZO-1 and Occludin was significantly decreased after DSS treatment, and *V. parvula* further intensified this impairment. In contrast, administration of MGQD substantially restored the structure of the colonic mechanical barrier (Figures 5A,B). These results were further confirmed by Western blot experiments (Figure 5C).

### 3.5 MGQD mitigates colitis symptoms in *Veillonella parvula*-Colonized mice

**FIGURE 6:**
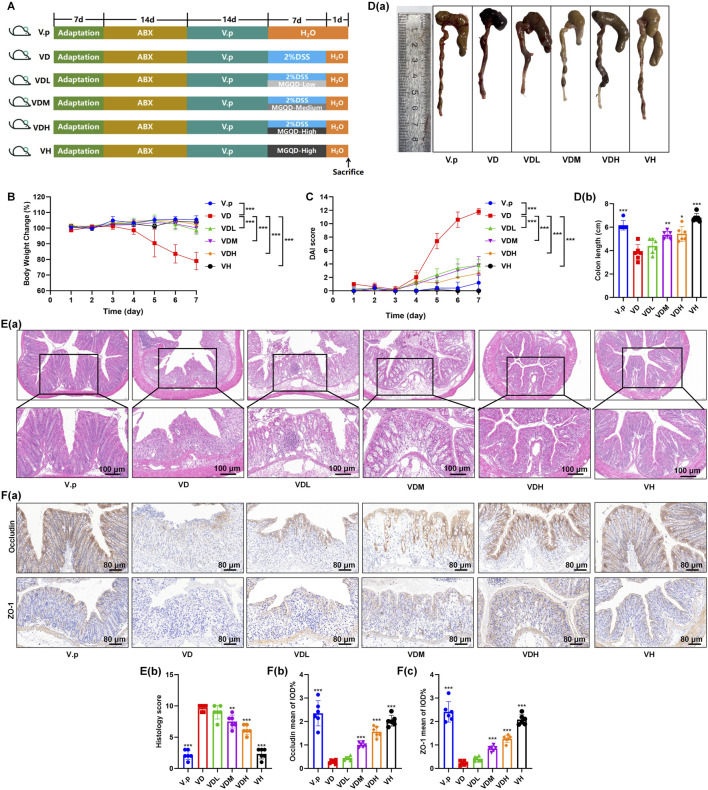
*V. parvula* aggravates DSS-induced colitis in pseudo-germ-free mice, while the MGQD mitigates this exacerbation. **(A)** Experimental timeline. **(B)** Body weight changes in mice. **(C)** DAI scores. **(D)** Representative colon tissue images and corresponding quantitative analysis. **(E)** Hematoxylin and eosin (H&E) staining of colon tissues and histopathological scoring. **(F)**The IHC staining in mouse colon. Data are presented as mean ± SEM. Group definitions: V. p: pseudo-germ-free mice that colonized *V. parvula*. VD: pseudo-germ-free mice that colonized *V. parvula +* DSS. VDL:pseudo-germ-free mice that colonized *V. parvula +* DSS + low-dose MGQD. VDM: pseudo-germ-free mice that colonized *V. parvula +* DSS + medium-dose MGQD. VDH: pseudo-germ-free mice that colonized *V. parvula +* DSS + high-dose MGQD. VH: pseudo-germ-free mice that colonized *V. parvula +* high-dose MGQD. The data are expressed as the means ± standard deviation (S.D.)(n = 6). *P < 0.05, **P < 0.01, ***P < 0.001 vs. the VD group.

**FIGURE 7:**
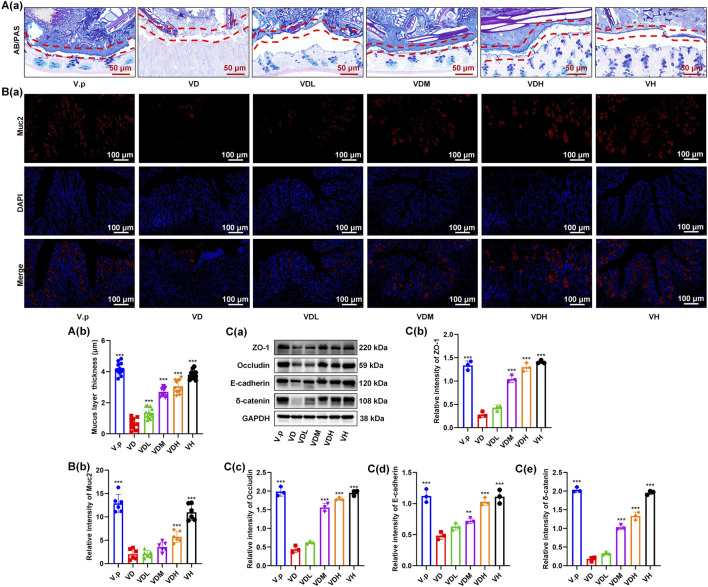
*V. parvula* exacerbates colonic barrier damage in DSS-induced colitis pseudo-germ-free mice, while the MGQD ameliorates this *V. parvula*-exacerbated damage. **(A)**The AB-PAS staining in mouse colon. **(B)** The IF staining of mouse colon for Muc2 (red) and DAPI (blue). **(C)** The Western blot of mouse colon. The data are expressed as the means ± S.D. (n = 6). *P < 0.05, **P < 0.01, ***P < 0.001 vs. the VD group.

We also examined the impact of *V. parvula* on the colonic mechanical barrier under inflammatory conditions in pseudo-germ-free mice (Figures 6A–E). Consistent with our previous observations, the expression of mechanical barrier markers E-cadherin and δ-catenin was significantly reduced (Figure 7C). Immunohistochemical analysis further showed that DSS treatment markedly decreased the expression of the barrier markers ZO-1 and Occludin, and *V. parvula* further exacerbated this impairment (Figure 6F). In contrast, administration of MGQD substantially restored the structure of the colonic mechanical barrier.

### 3.6 MGQD restores mechanical barrier integrity in *Veillonella parvula*-Colonized mice

We also examined the impact of *V. parvula* on the colonic mechanical barrier under inflammatory conditions in pseudo-germ-free mice. Consistent with our previous observations, the expression of mechanical barrier markers E-cadherin and δ-catenin was significantly reduced (Figure 7C). Immunohistochemical analysis further showed that DSS treatment markedly decreased the expression of the barrier markers ZO-1 and Occludin, and *V. parvula* further exacerbated this impairment (Figure 6F). In contrast, administration of MGQD substantially restored the structure of the colonic mechanical barrier.

### 3.7 MGQD ameliorates mucus barrier damage in *Veillonella parvula*-Colonized mice

Consistent with previous findings, in pseudo-germ-ree mice, *V. parvula* significantly exacerbated mucus barrier damage under inflammatory conditions, as evidenced by reduced mucus layer thickness (Figure 7A). Immunofluorescence analysis further confirmed that *V. parvula* mediated the downregulation of mucin Muc2 expression (Figure 7B). In contrast, MGQD treatment significantly restored mucus layer thickness (Figure 7A) and prevented the *V. parvula-*induced downregulation of Muc2 (Figure 7B), demonstrating the ability of MGQD to maintain mucin production during inflammation.

### 3.8 Baicalin and Puerarin Inhibit TNF-α-Induced Damage in Colonic Organoids

**FIGURE 8:**
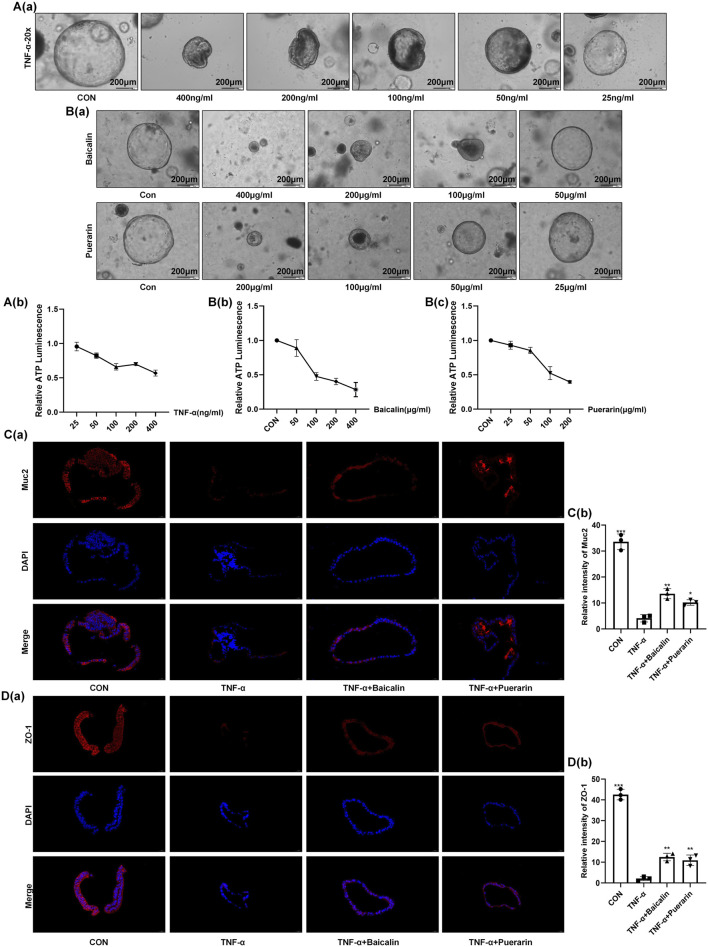
Baicalin and Puerarin Inhibit TNF-α-Induced Damage in Colonic Organoids. **(A,B)** Bright-field micrographs and statistical analysis of organoid viability. **(C)** The IF staining of organoid for Muc2 (red) and DAPI (blue). The concentration of baicalin and puerarin is 50 μg/mL. The concentration of TNF-α is 100 ng/mL. **(D)** The IF staining of organoid for ZO-1 (red) and DAPI (blue). The concentration of baicalin and puerarin is 50 μg/mL. The concentration of TNF-α is 100 ng/mL. The data are expressed as the means ± S.D. (n = 3). *P < 0.05, **P < 0.01, ***P < 0.001 vs. the TNF-α group.

This study used a colonic organoid model to assess the regulatory effects of MGQD’s active ingredients, baicalin and puerarin, on intestinal barrier function *in vitro* in order to better understand the drug’s mode of action. The findings showed that puerarin and baicalin considerably reduced the damage caused by TNF-α in colonic organoids (Figures 8A,B). Both elements improved intestinal barrier function by activating the expression of the tight junction protein ZO-1 and the mucin Muc2, according to immunofluorescence studies (Figures 8C,D).

## 4 Discussion

**FIGURE 9:**
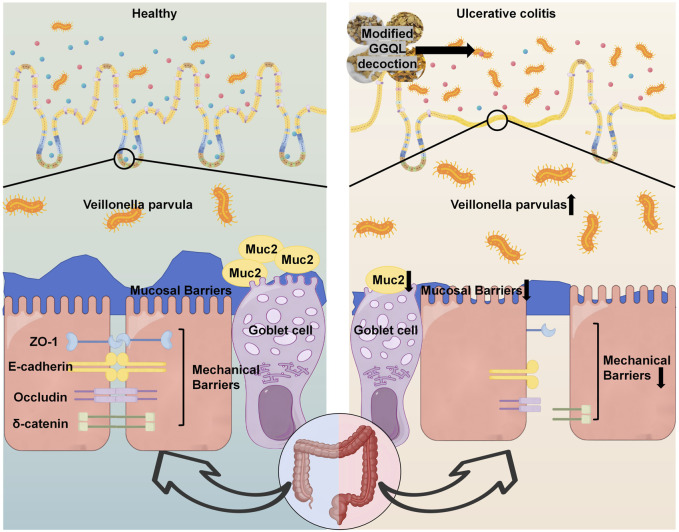
MGQD ameliorates Veillonella parvula-exacerbated ulcerative colitis via restoration of intestinal mucosal barrier function.

This study elucidates for the first time that *V. parvula* acts as a key pathobiont in the DSS-induced inflammatory milieu, exacerbating UC progression through the synergistic disruption of both the intestinal mucus barrier and the mechanical barrier (Figure 9). Histological analysis revealed characteristic pathological features in DSS-model mice, including submucosal edema, inflammatory cell infiltration, and crypt structural damage. Within this inflammatory context, nitrate enrichment drives a metabolic shift in *V. parvula* from fermentation to anaerobic respiration, promoting its proliferation. Also, *V. parvula* exacerbated colonic injury, manifested as increased disease activity index scores, pronounced weight loss, colon shortening, specific compromise of mucus layer integrity, impairment of epithelial barrier function.

Mucus barrier impairment is an early critical event in UC (van der Post et al., 2019). UC patients exhibit defects in this barrier, including reduced core components and aberrant permeability, which compromise its ability to segregate gut microorganisms (van der Post et al., 2019; Johansson et al., 2014; Matute et al., 2023). This study found that *V. parvula* further aggravates this barrier deficiency, potentially through mechanisms involving mucin degradation and effects on goblet cell function, establishing a vicious cycle of “barrier damage-microbial translocation-exacerbated inflammation”. Although LPS from *V. parvula* has been implicated in epithelial barrier disruption, its precise mechanistic pathways remain unclear (Zhan et al., 2022).

Regarding the mechanical barrier, tight junction proteins, including ZO-1 and claudins, are core structures maintaining epithelial integrity (Qiu et al., 2024; Mehandru and Colombel, 2021). This study found that *V. parvula* infection significantly downregulated the expression of tight junction proteins, disrupting intercellular connections and increasing intestinal permeability. Notably, the mucus and mechanical barriers are functionally interconnected: damage to the mechanical barrier can weaken the anchorage and stability of the mucus layer, while defects in the mucus barrier leave the epithelium directly exposed to luminal contents, further burdening the tight junctions (Sharma et al., 2018). *V. parvula*' concurrent disruption of both barriers creates a synergistic effect, significantly accelerating UC progression.

The core of TCM compound therapy lies in its multi-component, multi-target synergistic actions (Cheng et al., 2022). Our LC-MS/MS analysis identified 12 major chemical components in MGQD, preliminarily revealing the complexity of its material basis (Wang et al., 2023). Mechanistically, MGQD demonstrates multi-level, interconnected pharmacological effects: In terms of immunomodulation, it effectively inhibits the release of key pro-inflammatory cytokines such as TNF-α, IL-1β, and IL-21, and suppresses the activation of the NLRP3 inflammasome and the differentiation of γδT17 cells, thereby effectively regulating intestinal immune-inflammatory responses^[2^ (Ma et al., 2023). Regarding barrier repair, MGQD potently reconstructs the intestinal epithelial physical and chemical barriers by protecting goblet cells, promoting mucin MUC2 secretion, and upregulating the expression of tight junction proteins (Huang et al., 2024). Furthermore, its efficacy is highly dependent on the remodeling of gut microecology. Studies confirmed that MGQD modulates the overall structure of the gut microbiota and improves bile acid metabolism, effects validated by microbiota depletion and fecal microbiota transplantation experiments (Liu et al., 2024).

In summary, MGQD treats UC not through a single pathway but via a multi-faceted synergistic network mechanism that through anti-inflammatory and antioxidant effects, direct mucosal barrier repair, and regulation of the “gut microbiota-metabolism” axis, it ultimately collectively restores intestinal immune homeostasis. This provides a solid scientific basis for its clinical efficacy and aligns with the holistic regulatory characteristics of TCM.

## Data Availability

The data presented in the study are deposited in the NCBI repository, accession number PRJNA1418895.
