# Supplementary material for: Modified gegen qinlian decoction ameliorates Veillonella parvula-exacerbated ulcerative colitis via restoration of intestinal mucosal barrier function
Source: Front Pharmacol. 2026 Apr 21;17:1803816. doi: 10.3389/fphar.2026.1803816 (PMC13139156; doi:10.3389/fphar.2026.1803816)
Supplement: Supplementary file 1 [file Supplementaryfile1.docx]

**Supplementary Figure 1**


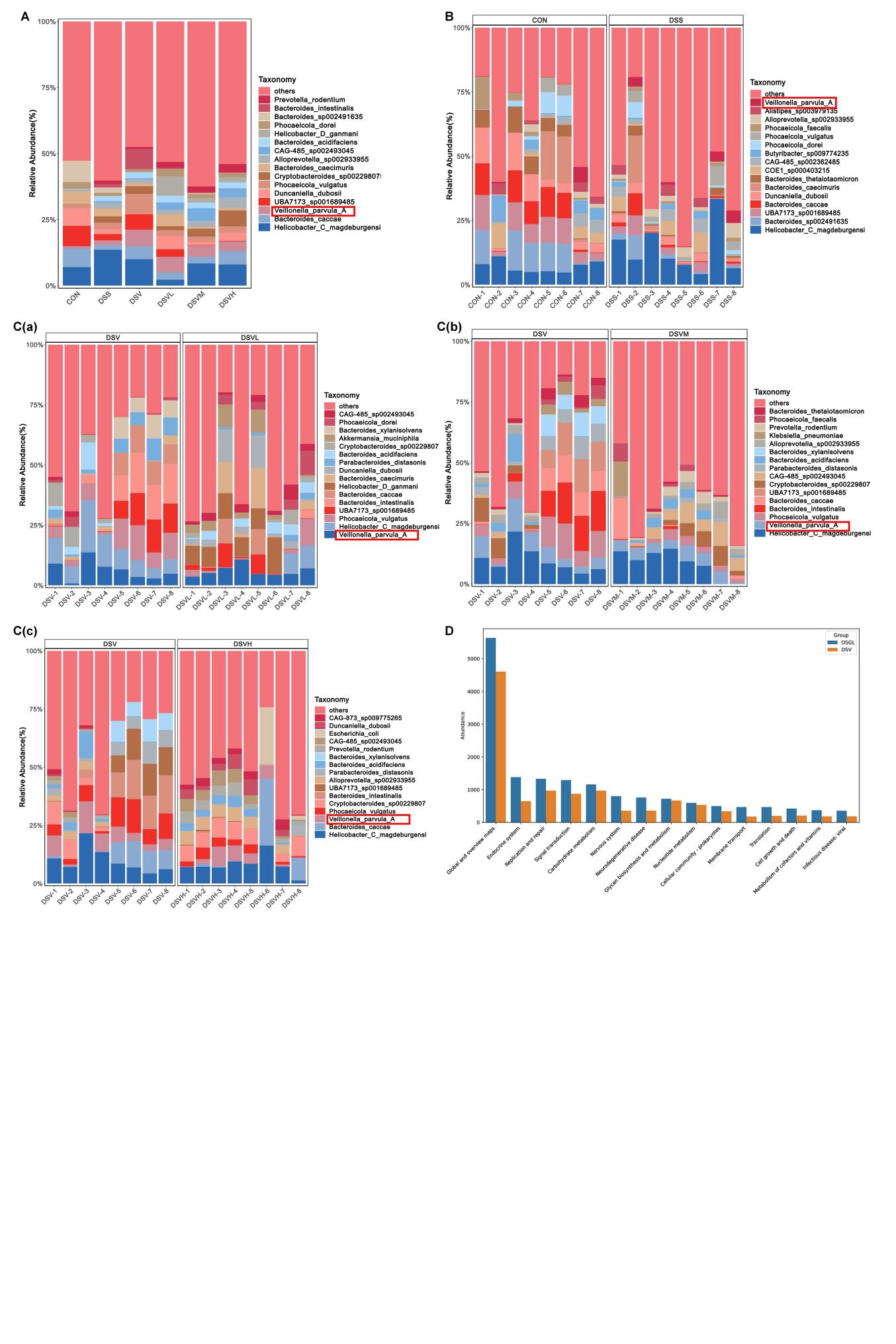


**Supplementary Figure 1**|(A-C): Column plot of species abundance of each sample at Species level. (D): The KEGG analysis of divergent microbiota. Data are expressed as mean ±SEM (n=8).

**Supplementary Figure 2**


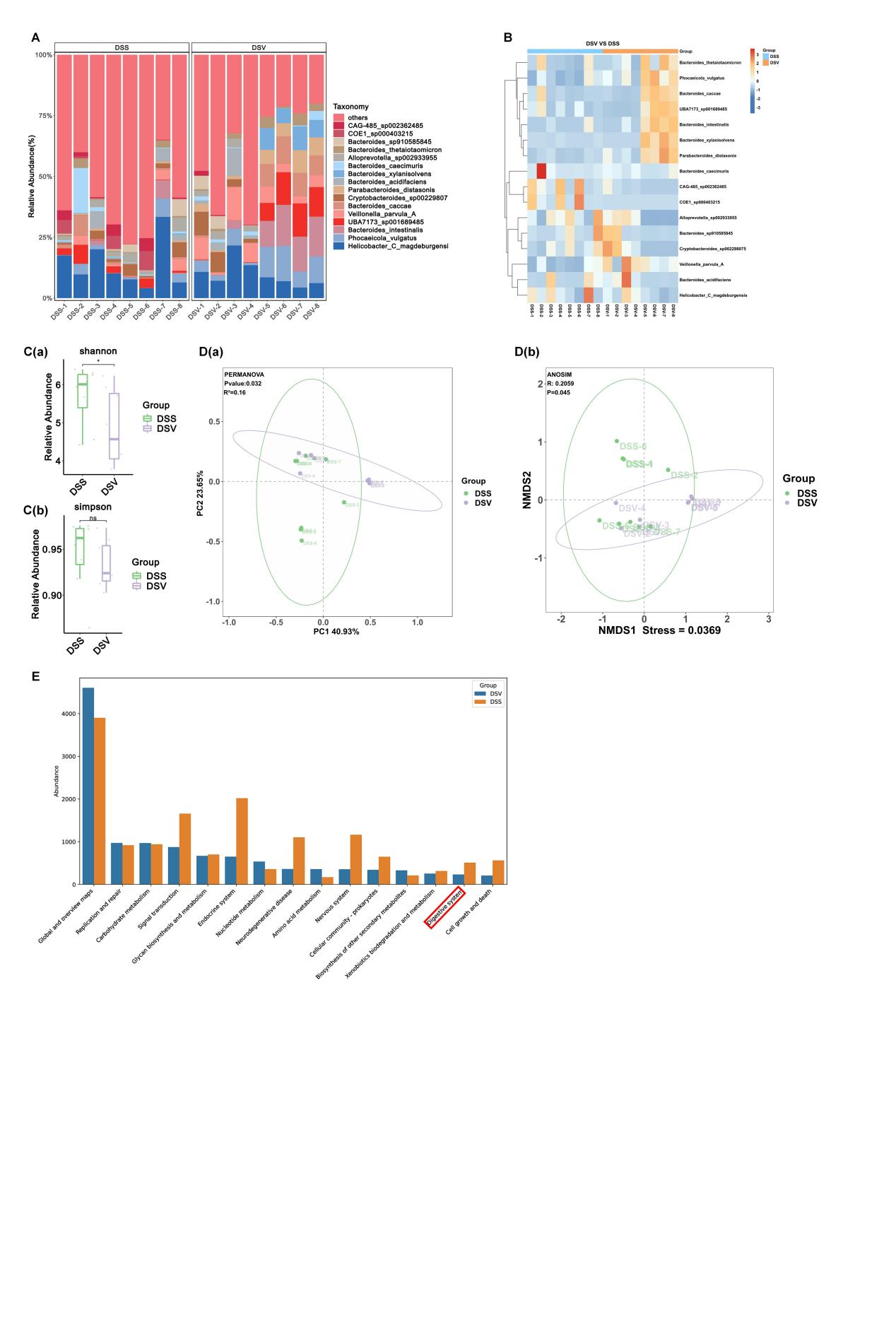


**Supplementary Figure 2**|(A): Column plot of species abundance of each sample at Species level. (B): Heatmap of species abundance of each sample at Species level.(C)The shannon index and simpson index. (D) The PCoA analysis and NMDS analysis. (E)The KEGG analysis of divergent microbiota. Data are expressed as mean ±SEM (n=8).

**Supplementary Figure 3**


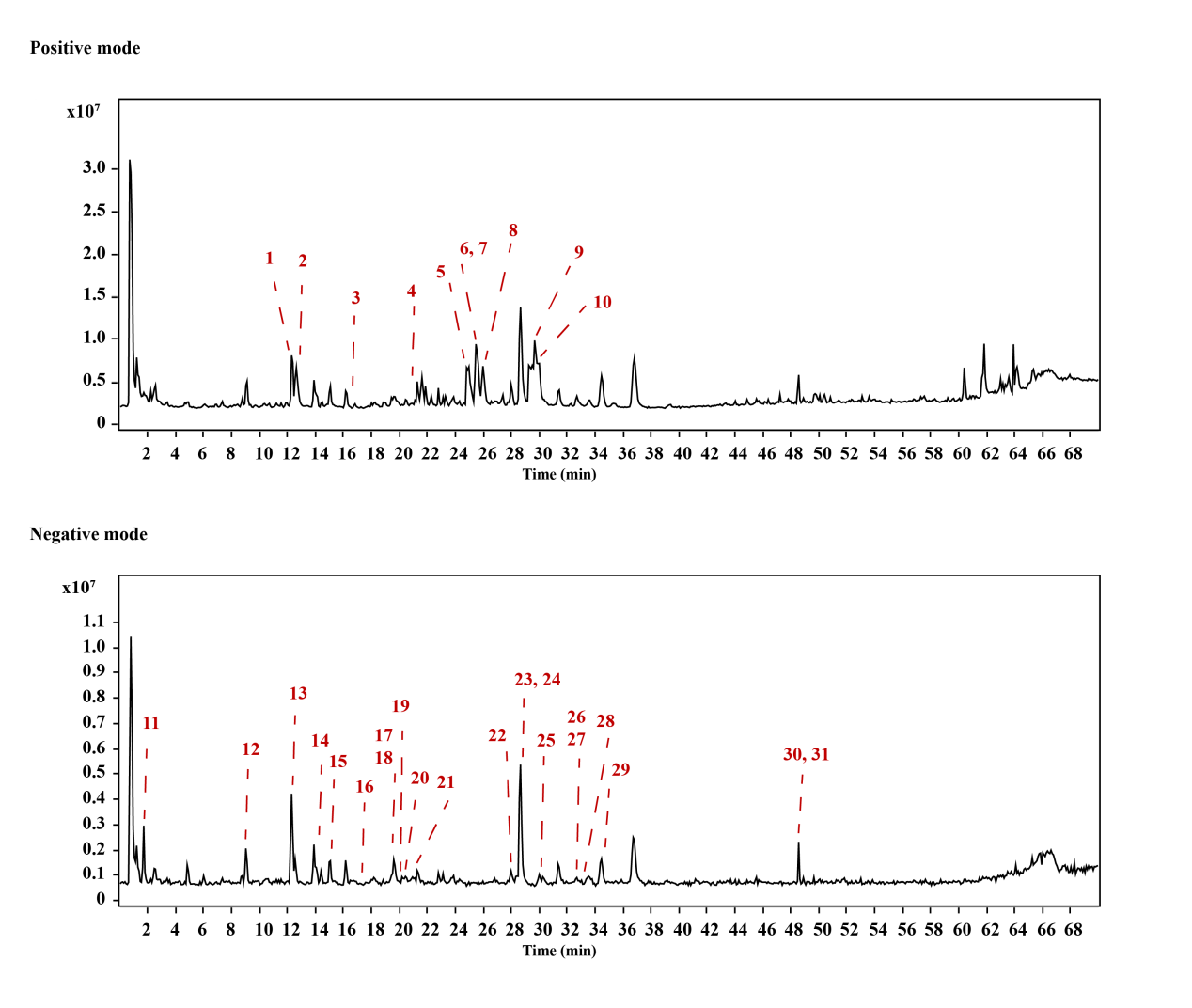


**Supplementary Figure 3**|Total ion chromatogram (TIC) of MGQD by UPLC-QTOF-MS/MS in positive and negative ion mode.1:Magnoflorine, 2:Methyl cinnamate, 3:Daidzin, 4:Glancine, 5:Coptisine, 6:Jatrorrhizine, 7:Columbamine, 8:Epiberberine, 9:Berberine, 10:Palmatine, 11:Gallic acid, 12:3’-Hydroxypuerarin, 13:Puerarin, 14:3’-Methoxypuerarin, 15:Puerarin apioside, 16:3’-Methoxydaidzin, 17:Daidzein-4’-glucoside, 18:Liquiritin, 19:Liquiritin apioside, 20:Ellagic acid, 21:Chrysin-6-C-arabinoside-8-C-glucoside, 22:Baicalin, 23:Liquiritigenin, 24:Daidzein, 25:Dihydrobaicalin, 26:Quercetin, 27:Luteolin, 28:Chrysin-7-O-β-glucuronide, 29:Oroxylin A-7-O-D-glucuronide, 30:Wogonin, 31:Glycyrrhizic acid.
